# Intelligent tutoring systems for word problem solving in COVID-19 days: could they have been (part of) the solution?

**DOI:** 10.1007/s11858-022-01396-w

**Published:** 2022-07-22

**Authors:** Javier del Olmo-Muñoz, José Antonio González-Calero, Pascual D. Diago, David Arnau, Miguel Arevalillo-Herráez

**Affiliations:** 1 University of Castilla-La Mancha, Albacete, Spain; 2 University of Valencia, Valencia, Spain

**Keywords:** Intelligent tutoring system, Problem-solving, Systematic review, Technology-based mathematics education, COVID-19, Socioeconomic level

## Abstract

The COVID-19 pandemic led to the lockdown of schools in many countries, forcing teachers and students to carry out educational activities remotely. In the case of mathematics, developing remote instruction based on both synchronous and asynchronous technological solutions has proven to be an extremely complex challenge. Specifically, this was the case in topics such as word problem solving, as this domain requires intensive supervision and feedback from the teacher. In this piece of research, we present an evaluation of how technology is employed in the teaching of mathematics, with particular relevance to learning during the pandemic. For that purpose, we conducted a systematic review, revealing the almost complete absence of experiments in which the use of technology is not mediated by the teacher. These results reflect a pessimistic vision within the field of mathematics education about the possibilities of learning when the student uses technology autonomously. Bringing good outcomes out of a bad situation, the pandemic crisis may represent a turning point from which to start directing the research gaze towards technological environments such as those mediated by artificial intelligence. As an example, we provide a study illustrating to what extent intelligent tutoring systems can be cost-effective compared to one-to-one human tutoring and mathematic learning-oriented solutions for intensive supervision in the teaching of word problem solving, especially appropriate for remote settings. Despite the potential of these technologies, the experience also showed that student socioeconomic level was a determining factor in the participation rate with an intelligent tutoring system, regardless of whether or not the administration guaranteed students' access to technological resources during the COVID-19 situation.

## Introduction

At the end of 2019, the Wuhan Municipal Health Commission (Hubei Province, China) reported a cluster of pneumonia cases in the city. It was later determined that the new virus SARS-CoV-2 was the cause of the so-called COVID-19 disease. Due to its alarming contamination rate and severity, on March 11, 2020, the World Health Organization declared it a pandemic (Cucinotta & Vanelli, 2020). In an attempt to contain the spread of the disease, millions of people across the world were confined to their homes in what has been unprecedented in recent decades. At the same time, educational institutions at all levels were closed down, affecting more than 1500 million learners around the world at the beginning of April 2020, a figure that corresponds to 91.3% of the total number of enrolled students, who were no longer able to attend their face-to-face lessons (UNESCO, 2020).

Many countries immediately opted for remote teaching and learning in response to this crisis, with a greater or lesser degree of success, influenced by their level of readiness to implement the necessary online systems (Toquero, 2020). At this point, it is convenient to distinguish between online or virtual education as it was called before the pandemic, from the urgent and sudden distance education that took place during the lockdown. Characterized by the temporary use of fully remote teaching solutions initially envisaged or conceived for face-to-face education, Hodges et al. (2020) called it Emergency Remote Teaching (ERT), and defined it as “a temporary shift of instructional delivery to an alternate delivery mode due to crisis circumstances” (para. 13). What everyone seemed to agree on at the beginning of the pandemic was the prediction of the negative impact of COVID-19 on students' learning, particularly in mathematics (Kuhfeld et al., 2020), with special concern for the expected substantial disparities between families (Burgess & Sievertsen, 2020) and an especially devastating impact in the case of disadvantaged students (Engelbrecht et al., 2020a, b).

When it comes to the sphere of mathematics education, despite all the efforts made to provide an appropriate response to the situation, there also seems to have been a difficulty in developing remote instruction based on both synchronous and asynchronous technological solutions, leading to learning losses in mathematics (Contini et al., 2021; Engelbrecht et al., 2020a, b). The experience of COVID-19 should serve to initiate a period of reflection and debate within the research community on the role that educational technology has played both in mathematical educational practice and in research in this field. Specifically, it is worth reflecting whether research in mathematics education has placed an excessive focus on some approaches or technological uses (in particular, teacher-mediated work) and marginalized others (in particular, autonomous student work), which could be an important factor in current educational settings and could have been more productive in remote teaching situations.

In that spirit, the purpose of this paper is enriching the debate about the use of technology for learning mathematics in post-COVID times. The first aim is to identify where the attention has been focused on research in technology-based mathematics education and what influence this could have had on the response provided from the community of mathematics educators during ERT. The second aim is to describe the potential of intelligent tutoring systems (ITS) in the field of problem solving and to provide an example of the use of an ITS in the teaching of mathematics during ERT. Both of these aims lead to further reflections on how technology has been used in mathematics education in recent years.

## Research on the use of technology in mathematics education: the role of teachers and students

The arrival of computer equipment in homes at the beginning of the 80´s, as a consequence of the miniaturization of the components and the reduction of the price, directed the attention of the research community to the possibility of implementing programs that emulated the actions of a human teacher, giving a possible economical solution to Bloom’s (1984) well known 2-sigma problem. In the case of the teaching and learning of mathematical problem solving, some proposals suggested the use of computer applications based on Artificial Intelligence (AI) (Fey, 1989).

But at that time, AI was not only identified as a discipline that could revolutionize the practice of mathematics education. In the special issue commemorating the 25th anniversary of the *Journal of Research in Mathematics Education*, Schoenfeld (1994) predicted that the use of AI would allow the creation of methods, within the area of mathematics education, that could possibly be considered scientific in the manner of the natural sciences. Thus, “the successful implementation of a problem-solving program provided empirical proof that particular theoretical ideas about thinking really 'work'” (pp. 707–708).

However, the optimism associated with the potential of technology in general, and artificial intelligence in particular, did not seem to be demonstrated in the development and experimentation of technological tools for teaching mathematics. Thus, in another paper in the same issue of that journal, Kaput and Thompson (1994) pointed out a significant absence of articles focused on technology-related research. As a possible cause, these authors indicated a passivity of the educational community in mathematics education, which led to accepting technology designed for other purposes (e.g., calculators, spreadsheets, computer algebra systems or programming languages) uncritically, and assuming the development of these technological tools to be a matter for specialists outside the area.

Coinciding with the previous situation, at the beginning of the 90’s the expectations placed on AI in general, associated with the development of increasingly powerful computers, vanished as a result of the limited observed capacity of intelligent environments to be able to provide assistance to students in a one-on-one situation (McArthur & Lewis, 1998). Within the area of mathematics education, this caused a stagnation in AI studies in mathematics education, which was probably exacerbated by the characteristics of mathematical knowledge. In this way, Balacheff and Kaput (1996) argued that the developers of AI applications paid little attention to the role of the teacher, and that their design was based on a separation between declarative and procedural content and between the content of the interface and the control and inference structures. For these authors, these separations were problematic, since they implied a difficult communion with the demands of the teaching of mathematics such as the following: the use of multiple representation systems of mathematical knowledge, the need to take into account the student's intention, or the complexity of attending to the negotiation of meanings between the teacher and the students.

This loss of interest in the development of applications based on AI coincided with the development of tools adapted to the teaching of mathematics from general purpose technological tools (e.g., graphical calculators, micro-worlds, or educational computer algebra systems). Furthermore, the development of operating systems with graphical environments opened a new point of attention in the teaching of mathematics, focused on the possibility of making simultaneous expressions of different representation systems (e.g., dynamic geometric environments). On the other hand, the arrival of the Internet made it possible to develop applications that favour collaborative work in both synchronous and asynchronous situations. In these cases, the management of the teaching and learning process was left to the teacher and, indirectly, this could have provoked researchers to focus on the possibilities of using technology coupled with human teaching. The result of all of the above could be summarized in a pessimism in mathematics education research regarding the possibilities of reproducing human teachers’ actions through AI applications, as well as the possibilities in the field of teaching for learning to occur in situations in which students used technology autonomously. This point of view was clearly stated in the Technology Principle (NCTM, 2000): “Technology does not replace the mathematics teacher. When students are using technological tools, they often spend time working in ways that appear somewhat independent of the teacher, but this impression is misleading” (p. 26). But has this perspective been a dominant one in mainstream mathematics education research? To answer this question, we conducted a systematic review on the use of technology in mathematics education. The review was aimed at determining whether the research in technology-based mathematics education has been focused on developing and assessing solutions not centred on autonomous student work, which have been of little use in the situation caused by COVID.

There have been several review articles and meta-analyses on the roles of technology in mathematics education, such as on its effects on students' mathematics achievement (e.g., Li & Ma, 2010; Verbruggen et al., 2021), on its usage in the classroom (e.g., Bray & Tangney, 2017), and on its effects on students’ motivation and attitudes (e.g., Higgins et al., 2019). These studies show that re-examination of the use of technology in mathematics education is not a new area of research, but each of these works classifies technology use in different ways.

In an attempt to unify all these classifications, the second-order meta-analysis by Young (2017) categorized prior meta-analyses into the following three categories according to the instructional function of technology: computation enhancement technologies that make use of calculators; presentation and modelling enhancement technologies, involving mathematics-specific software applications such as dynamic geometry software or virtual manipulatives; and instructional delivery enhancement technologies that would allow teachers to individualize instruction, such as computer-assisted instruction (CAI) or computer-based instruction.

Despite this diversity of categorization formats, it is noteworthy that the hierarchies employed in earlier research to define interventions do not always consider the individualization of the instruction, and even when they do (e.g., Li & Ma, 2010; Young, 2017), there has not been a place in any of them (to the best of our knowledge) for the ‘standalone’ type of instruction that would allow students to keep learning autonomously. Therefore, we attempted to examine systematically previous empirical research that involved the use of technology in mathematics education, contemplating whether this use was isolated in terms of teacher intervention for educational aspects. The motivation behind conducting a systematic review using this categorization was to examine to what extent the investigation into this matter has been focused on the need for a teacher-mediated process rather than on the use of systems capable of emulating the teacher's action, which could be very useful in situations with the characteristics of the COVID pandemic. In particular, in this analysis we aimed to determine whether the empirical research on the use of technology in mathematics education has been more oriented to a non-standalone use of technology.

The systematic review drew from recently published scientific literature and included empirical studies on the use of technology in mathematics education, within the framework of student autonomy. For the proper conduct of systematic reviews, we followed the guidelines set forth in the Preferred Reporting of Items for Systematic Reviews and Meta-Analyses (PRISMA) procedures (Moher et al., 2009; Page et al., 2021).

The literature search focused on journals according to the following criteria: (1) they were indexed up to 2020 in the Social Sciences Citation Index (SSCI) of the Web of Science database in the *Education & Educational Research* category; and (2) they were specialized in the publication of research papers mathematics education. The first criterion yielded a total of 265 journals, while the second reduced the number to a total of 10 journals, which are listed as follows: *Journal of Mathematics Teacher Education, School Science and Mathematics, Eurasia Journal of Mathematics Science and Technology Education, Educational Studies in Mathematics, Journal of Research in Mathematics Education, International Journal of Science and Mathematics Education, Mathematical Thinking and Learning, International Journal of Mathematical Education in Science and Technology, Revista Latinoamericana de Investigación en Matemática Educativa,* and *Enseñanza de las Ciencias*.

Specifically, we conducted a search of articles limited to the journals on the list using the tag ‘IS’ (ISSN/ISBN) in combination with the terms 'Technology' AND ‘Mathematics’ in the tag ‘TS’ (Topic), which allowed us to discriminate categories from titles and abstracts. This search, which we conducted in November of 2021, yielded 817 references, a large number of results that provided us with a broad picture of the topic's scope.

Before proceeding with the selection of studies from the initial search, we defined the inclusion and exclusion criteria as follows. (1) The articles should report empirical studies. Consequently, review studies, theoretical papers or classroom notes were excluded. (2) The studies had to evaluate at least one technological tool with which the study participants completed mathematical activities. An example of exclusion based on this criterion would be a study that used eye-tracking technology to assess externally what strategies students used while solving problems. (3) The studies had to be focused on the teaching and learning of mathematics. Studies framed in other subjects such as science, technology, etc., were discarded. (4) The studies had to offer enough information to enable us to determine the role that technology played in the learning process.

The application of the search criteria allowed us to identify an initial set of 817 articles. Next, during this screening process based on an analysis of the content of the articles, 643 articles were discarded for different reasons, including the following: because it was a different domain than mathematics (n = 310); because it was not an empirical study (n = 137); because it did not evaluate a technological tool used by the participants (n = 186); or because it did not offer enough information (n = 10). Among the 174 papers that met the inclusion criteria, we distinguished between two types of studies, namely, those in which the participants did not work autonomously, that is, they worked under the guidance of the teacher/instructor in one way or another (n = 164), and those in which the participants made a standalone use of technology, in which the teacher/instructor did not intervene except in answer to technical questions, if applicable (n = 10). Thus, only 10 of the 174 studies investigated a standalone use of technology, and the tool used in no case was an ITS. The only case where an ITS was used was in a non-standalone instruction situation.

The most remarkable stages in which those 174 studies were conducted include kindergarten (n = 5), elementary school (n = 15), secondary school (n = 36), higher education (n = 37), pre-service teachers (n = 39), in-service teachers (n = 32), or students with special needs (n = 3). Moreover, the different investigations analysed various dependent variables, with learning of mathematics being the most common (n = 76). Concerning the types of technological set-up, in order to characterize the 174 studies, we identified that the focus in those papers stands on the following types: the use of computer algebra systems (n = 12); calculators and graphical calculators (n = 17); coding-based tools (n = 6); dynamic geometric software (n = 47), technological tools with general purpose, such as spreadsheets or statistical software (n = 33); educational-oriented technologies but not addressed to mathematical content, such as educational platforms or remote response systems (n = 28); mathematic learning-oriented technologies, such as *Algebra Arrows* or *TinkerPlot* (n = 25); video-based platforms for general purpose (n = 21), internet-based tools, such as generic videoconference software or blogs (n = 11), and ITS (n = 1). It is important to mention that some of the analysed studies presented mixed technological configurations, so they were included in more than one of the above-mentioned categories. Finally, it is worth noting that the qualitative research method was the most widely used (n = 101), followed by quantitative research (n = 49) and mixed research (n = 24).

In particular, examining the 10 studies involving a technological standalone-use we found the following results: 2 were addressed with non-educational-oriented technology (one used virtual and augmented reality and the other used a recommendation system for geometry activities); 3 were addressed with mathematic learning-oriented technology (*CLIPS-Critical Learning Instructional Paths Supports-*, *PSADRI system-Problem-solving Assessment, Diagnosis, and Remedial Instruction* and *Teaching and Learning Integrated System SIENA*); 3 were conducted with dynamic geometric software (*GeoGebra or JSXGraph*); and 1 used coding-based tools (*Building Blocks* software suite). Table 1 provides detail for the standalone-instruction studies included in this review.

**Table 1:** Characteristics of standalone-instruction studies

Paper	*n*	Academic level	Research design	Math domain	Technology	System feedback	Major findings
Dallemole et al. (2014)	10	Higher education	Qualitative	Analytical geometry	SIENA	Validation feedback	The system proved to be efficient, contributing to the identification of the individual difficulties of the students, who showed better results in the subsequent tests
Foster et al. (2016)	243	Kindergarten	Quantitative	Early numeracy and geometry	Building Blocks	N/A	Children from low-income backgrounds improved numeracy and applied problems achievement after using Building Blocks
Hsiao et al. (2017)	153	Secondary school	Quantitative	Problem solving	PSADRI	Validation feedback	Students achieved greater learning performances in mathematics, improved problem-solving skills and increased their interest in mathematics learning
Jacinto and Carreira (2017)	1	Secondary school	Qualitative	Geometry problem solving	GeoGebra	N/A	The analysis of the student’s work with GeoGebra as a complement to her mathematical skills in solving geometrical problems, offers evidence of the efficiency of a techno-mathematical approach to achieve the solution of a problem
McCulloch et al. (2020)	2	Pre-service teachers	Qualitative	Conception of function	GeoGebra applet	No feedback	Based on the worked with the applet, it was demonstrated that one's conception of function is influenced by their previous experiences with function and the contexts in which they used it
Roca-González et al. (2016)	31	Higher education	Quantitative	Spatial abilities	Virtual and augmented reality	N/A	Engineering students autonomously performed a short virtual and augmented reality course, resulting in the significant improvement of their spatial abilities
Ross and Bruce (2009)	273 / 307	Secondary school	Quantitative	Fractions	CLIPS	Validation feedback	The effectiveness of five sets of activities (CLIPS) was evaluated in two experiments (the second being a replication of the first); the findings suggest that the students who completed the full sequence improved their understanding of fractions
Soldano et al. (2019)	20	Secondary school	Quantitative	Geometry	JSXGraph	Validation feedback	The implementation of an inquiry-based game in GeoGebra first (not automated) and in JSXGraph later (automated), helped students to develop certain forms of strategic reasoning
Su (2017)	94	Elementary school	Quantitative	Geometry	ALPRS	Feedback*	ALPRS benefits geometry-learning outcomes by providing distinct learning unit recommendations to students with different learning styles
Yoon et al. (2014)	1092	Higher education	Qualitative	N/A	Recorded lectures	No feedback	The results show that a large proportion of respondents considered recorded lectures unnecessary for their needs, which could be fulfilled by attending the live lecture (which also provides potential for interaction). A similar rate of participants provided reasons for combining live and recorded lectures (e.g., complementary features)

*N/A*, not available; Feedback*, the article does not provide enough information to classify the features of feedback

We may conclude from this analysis that research on mathematics in technology-based situations without teacher intervention is almost non-existent. Furthermore, the analysis of the distribution by educational stages of the reviewed articles reveals that a large part of the research concerning technology in mathematics is carried out with either pre-service or in-service teachers and that studies on compulsory education are underrepresented. An important point to note is that the development of applications from the area of mathematics education has been scarce and that most of the studies analysed the potential of general-purpose tools or educational technologies not centred on mathematics.

In particular, the attention to the development of ITSs capable of emulating human teachers, which was part of the concerns of the mathematics education research community during the 1980s, has not had continuity. In the next section, we reflect on the role of mathematics education in the development of intelligent tutoring systems devoted to word problem solving and we offer, as an example of its potential, an intervention carried out with an intelligent tutoring system during confinement.

## The potential of AI in mathematics education: the case of HINTS

An ITS is an AI application that aims to provide immediate and customized instruction, or feedback, to learners, usually without any intervention from a human teacher (Psotka et al., 1988). Some of the affordances that ITSs always incorporate, with the goal of being sensitive to the singularities of individual learners, are active student learning, interactivity (by systematically responding to the actions of the student), adaptivity (by presenting personalized information) and feedback (on the student’s performance) (Graesser et al., 2018).

The value of the contribution of ITSs to education is attested by several literature reviews and meta-analyses. In an extensive review comprising of 248 studies, Van Lehn (2011) compared the effectiveness of human tutoring, computer tutoring and no tutoring. This author concluded that the effectiveness of these learning environments had reached such a point that, although an ITS should not be used to replace a whole classroom experience, they could be an option as effective as one-on-one human tutoring, especially in STEM subjects. In this regard, the work of Van Lehn (2011) on the relative effectiveness of different tutoring systems enabled this author to infer from the reviewed studies that one-to-one human tutoring was the most effective approach, far surpassing large-group instruction. That being said, the author also underlined that the effect of human tutoring was lower than previously believed, and, in this regard, ITSs could be a competitive and suitable option, given their good cost–benefit ratio and considering that they are practically as effective as human tutoring.

Several studies concerning ITSs have also been carried out with a focus on mathematics education. Regarding this matter, Steenbergen-Hu and Cooper (2013) conducted a meta-analysis of 34 studies on K-12 students' mathematical learning through ITSs, concluding that ITSs had no negative effect and, at best, a small positive effect. Other notable findings from this work were that the effects appear to be greater when they involved general groups of students rather than low achievers, and were also greater when the ITS intervention lasted for a short time, or one semester at most, compared to when it lasted for one school year or longer. In a similar fashion, in the meta-analysis carried out by Kulik and Fletcher (2016) investigating ITSs’ effect on K-12 mathematics performance, higher effect sizes were found when it came to elementary and high school mathematics contexts.

In the specific case of the development of ITSs aimed at teaching and learning problem solving, the system needs to be able to evaluate the solution process (step-based systems), and not just the final result (answer-based systems), and to identify relevant aspects of a student’s actions that allow the system describing the strengths and weaknesses of a student in the mathematical content at hand. To describe the potential of an ITS in assessing the solving process, Van Lehn (2011) introduced the term granularity as “the amount of reasoning required of participants between opportunities to interact” (p. 202), i.e., the larger the user interface’s grain size, the more reasoning is required from the user in each interaction. Obviously, from an empirical point of view, the educational potential of step-based systems is greater than that of answer-based systems compared to situations in the absence of human tutoring (du Boulay, 2016).

The implementation of ITS prototypes aimed at teaching and learning both arithmetic and algebraic word problems solving must meet the following two requirements: (1) supervising the validity of student’s solutions, and (2) collecting information about the student's performance. The first requirement must allow the identification of successes and errors and provide help during problem resolution. The second requirement allows the ITS to model the student’s knowledge about problem solving and use this knowledge as a basis for the educational decisions that the system should make. The need to monitor the solving process requires the design of systems with a fine granularity, which, at the same time, are flexible to the decisions made by the solver. In the case of the arithmetic solving of one-step word problems, combining these two requirements is easier, since these problems are usually associated with a single solution path (a single structure of relationships between quantities) and, as a consequence, the amount of reasoning required to solve the problem matches that needed between two interaction opportunities. In multi-step problems, the solving process requires more than one interaction and there is the possibility that the same multi-step problem can be solved via different solution paths (several structures of mathematical relationships between quantities). In this case, in order to produce a fine granularity, it is necessary to provide the ITS with the ability to determine the validity of a step, and to identify the solution path, out of many available ones, that a student is following, in order to articulate hints based on the status of the solving process. Combining these two requirements has been a complex matter that requires, as Balacheff and Kaput (1996) point out, a separation between declarative and procedural content and the provision to the ITS of a representation system of the mathematical content that allows the translation of the (diverse) productions of the solvers to a more abstract representation system.

Technological proposals have avoided this complexity by limiting the degrees of freedom during the solving process or reducing the system's ability to determine the validity of the actions performed by solvers. For example, *AnimalWatch* (Beal, 2013) can verify arithmetical solving process, but the systems force the solver to follow a specific path; *ANIMATE* (Nathan, 1998) allows students to solve problems algebraically, but is unable to monitor their actions. The intelligent tutoring system HINTS (Hypergraph-based INtelligent Tutoring System) is a step-based system with a fine granularity. This system allows the user to solve word problems both arithmetically and algebraically (Arnau et al., 2013) and is capable of validating the solver’s actions according to the chosen solving path and the state of the solving process (Arnau et al., 2014). The ITS can provide recommendations on-demand by offering both general and task content information according to the solution path that the student is hypothetically following. To achieve this, when a problem has several solving paths, the system identifies the path with the highest proportion of mathematical relations used, by keeping track of which relations have already been used in each possible solving path.

**Fig. 1:**
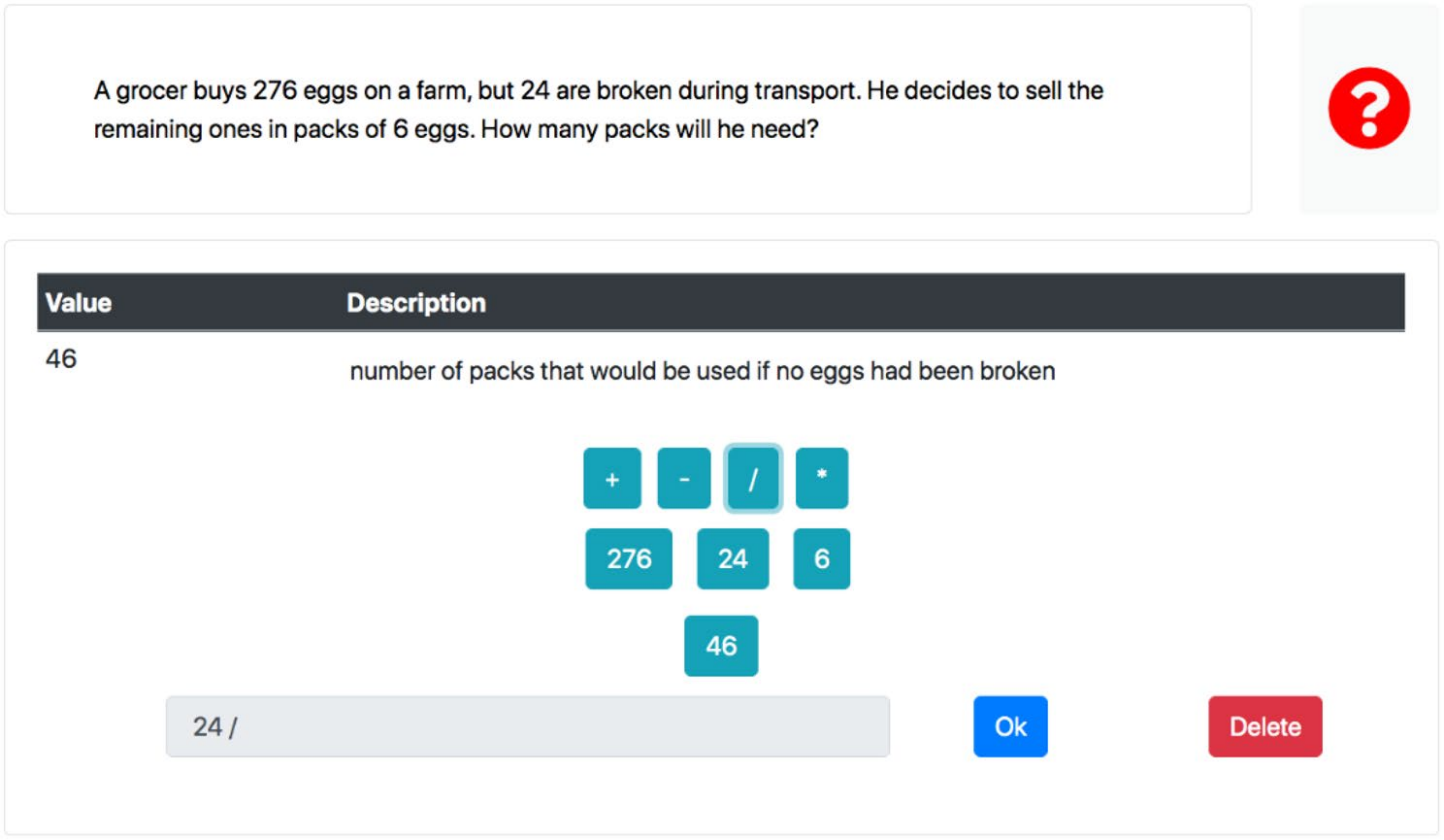
Example of a user solving an arithmetic word problem in HINTS

Currently, the application is accessible from any computer with a browser connected to the Internet. The interface that HINTS shows to the user is configurable in order to adapt it to different didactical or experimental intentions and the level of the information provided in on-demand hints is also configurable (del Olmo-Muñoz et al., 2022). In the case of arithmetical problem solving, a possible interface represents the quantities through a calculator-like component (Fig. 1). This component contains a button for each of the known quantities needed to solve the problem, as well as buttons for the four basic operations. The student can calculate the unknown quantities by entering arithmetic expressions using the calculator-like component. These expressions are validated by the system. When the system determines that a student's action was correct, it automatically calculates the result of the operation, generates a new button with the value of the just computed unknown quantity, and displays a description of the quantity and its value. Otherwise, the system displays an error message. All the interactions between the solver and the system are stored in a database.

## The potential of ITSs: an example in the COVID-19 situation

In light of the previous sections, it seems clear that ITSs are a possibility worth considering as they could lead to a cost-effective option for one-to-one instruction when compared to a teacher-to-student solution. However, there are other factors, such as the socioeconomic variables, that need to be considered when analysing the full picture of these events in a lockdown context. Socioeconomic status (SES) plays a major role in individuals’ lives, as growing up in poverty has been widely shown to have detrimental developmental implications (Bradley & Corwyn, 2002). Low SES also has a heavy negative impact on school achievement (Brooks-Gunn & Duncan, 1997), especially when exposure to these conditions is produced during the stage of early childhood (from birth to 8 years old) (Duncan et al., 1998). This issue has been investigated in depth, using different large-scale evaluations such as the OECD’s Programme for International Student Assessment (PISA) survey (Lagravinese et al., 2020) or the IEA’s Trends in Mathematics and Science Study (TIMSS) (Broer et al., 2019), sometimes focusing specifically on mathematics (Reddy et al., 2019), and there is ample evidence that some of the problems in education are due to socioeconomic gaps (Lagravinese et al., 2020).

Children from low-income families are also more likely to struggle with engagement in school (Jensen, 2013). Given that the participation of students in free or reduced-price lunch programs is an indicator of the SES of their households, a study with more than 81,000 students found that those students who were eligible for free or reduced-price lunch programs reported lower levels of engagement than students who were not eligible for those programs (Yazzie-Mintz, 2009). Jensen (2013) offered seven differences between middle-class and low-income students (health and nutrition, vocabulary, effort, hope and growth mind-set, cognition, relationships, and distress) which could explain this phenomenon, and outlined different strategies that teachers could apply in the classroom to mitigate some of the damaging impacts of poverty, including positive, prompt, actionable, and task-specific feedback.

Specifically, the effect of low SES is clearly observed in the possibilities of families with low economic resources to provide their children with extracurricular support. In regard to technology, Bond and Bedenlier (2019) discussed how the students’ family SES affects their capacity to afford devices and access the Internet, and how this, in turn, affects their children’s attitudes towards technology. To bridge the digital gap, they emphasized the need for making low-cost technology available to students and families, for example by developing institutional equipment loan programmes. Vekiri (2010) examined the correlation between socioeconomic level and elementary students’ information and communication technologies (ICT) beliefs, concluding that students from low-income homes appear to have lower chances to develop ICT skills since they are less likely to have access to a computer and the Internet at home. In addition, these students’ families are also less likely to know how to guide their computer use in a way that promotes the development of their ICT literacy.

As predicted by Burgess and Sievertsen (2020), all these educational inequalities have been exacerbated by COVID-19 across different countries (Bayrakdar & Guveli, 2020; Engzell et al., 2021; Putra et al., 2020) and it seems likely that this will also leave legacies in the long term (Blundell et al., 2020). One of the reasons seems to be the significant disparity concerning how capable parents believe they are of helping their children, with lower educated parents being less confident of their ability to support their children, even in primary education (Cullinane & Montacute, 2020). Many parents were even unable to devote time to educational activities at home during the lockdown. As a result, children from wealthier households spent 30% more time at home learning than children from impoverished homes (Andrew et al., 2020).

In this context, in early 2020 we were planning to conduct an experiment to evaluate the effectiveness of students using the system HINTS (Arnau et al., 2014) to solve arithmetical word problems rather than solving them on paper. Although, due to the pandemic, the educational administration decided to confine students at home before we started the experiment, we opted for adapting it to the new scenario. Our primary goal was to give students the opportunity to continue practising mathematics in that unusual situation. Thereby, the experiment allowed us to analyse the potential of an ITS for the teaching of arithmetic word problems in a standalone situation. In addition, the adaptation of the experiment to a ERT situation made it possible to address new research objectives. Specifically, we attempted to address the following questions in a situation that simulated the completion of homework where all students had been provided with computers and internet access: (1) Is attendance at remote standalone word problem solving instruction supervised exclusively by HINTS in the COVID-19 ERT situation conditioned by the income level of the students? (2) Is there an income gap in students’ performance in remote standalone word problem solving instruction supervised exclusively by HINTS during the COVID-19 ERT situation?

### Intervention

The intervention aimed to determine the degree of correlation between students’ SES and their academic achievement in mathematics after participating in supervised remote standalone learning activities. It may, however, be considered as an exploratory study in the sense that the pandemic scenario in which it was conducted is completely novel, and it will help us to better understand this new social situation. National studies typically use school-level metrics such as free and reduced lunch-eligibility as a proxy for poverty status (Avvisati, 2020), as it is often the best available measure (Rutkowski et al., 2018). Therefore, for this study we chose public information from the school meal service aid program as an indicator of the students' SES.

The participants in the research were 133 students (70 male and 63 female) from elementary education years 5 and 6 in a public primary school in Castilla-La Mancha, a region of Spain. It is worth noting that all of the students who took part in the study had the same access to resources in order to complete the activities, as the educational administration supplied the required technological means (laptop and internet connection) to the families that needed it.

In the experimental phase we used the intelligent tutoring system HINTS. The intervention was carried out using an interface which only allows the user to solve the problem in an arithmetical way. Given the COVID-19 situation, it was decided to prevent students from requesting aids on-demand to avoid the possibility that they would solve problems based exclusively on the messages offered by HINTS. Students could drop one problem at any time and automatically move on to the next one. When a student successfully solved a problem, the system provided a short report and then offered the next problem.

The problems used in the intervention were in the style of the problem in Fig. 1. The problem with the shortest solving path had two steps, while the problem with the longest path had six steps. The known and unknown quantities in all cases had exclusively positive integer values.

The first step of the procedure was to prepare an easy-to-follow document for students with instructions on how to proceed in each session and what devices they could use (computer or tablet). The document also included an email address to contact in case of any technical problems and a link to the ITS website, as well as a link to a short video tutorial (about 7 min long) in which the students were taught how to use the ITS, including how to log in with their personal credentials, use the basic controls, get help with problem quantities and, in general terms, interact with the interface. An explicit statement was included at the end of the document which emphasized how important it was for the students to try their best and not resort to the help of their parents or siblings.

The intervention with HINTS lasted four sessions over a 4-week period, one session per week. Each participant individually and asynchronously accessed HINTS with their personal user’s information to complete the activities. Students had one week to complete each session, which was made up of a collection of 6-to-10 word problems with the above-mentioned characteristics each. A total of 28 word problems were administered.

### Results

As regards the students’ attendance at virtual lessons, in order to draw a comparison between low-income students and non-low-income students we used the Pearson’s chi-square test. Table 2 shows the number of students who attended the first virtual lesson and any of the scheduled virtual lessons disaggregated by the participants’ income. According to these data, there was a significant association between income level and student attendance, either at the first scheduled lesson (*χ*^*2*^(1) = 6.06, *p* = 0.0138) or at any of the four lessons (*χ*^*2*^(1) = 3.87, *p* = 0.0492). This seems to represent the fact that, based on the odds ratio, the odds of students not attending the first lesson, or any of the lessons, were 2.82 and 2.29 times higher, respectively, if they were low-income students, than if they were non-low-income participants.

**Table 2:** Students’ attendance at virtual lessons

Income	Students’ attendance at the first virtual lesson		Students’ attendance at any virtual lesson	
	Yes	No	Yes	No
Non-low	66	38	74	30
Low	11	18	15	14

Concerning the second research question, first, we analysed the participants’ grades comparatively in the subject of mathematics in the second term between non-low-income and low-income students. These grades were computed taking into consideration the school period prior to the closure due to the pandemic. Therefore, only face-to-face activities were evaluated. For all the students who were invited to take part in the study, it was observed that the average low-income students’ academic performance (M = 6.11, SD = 2.08) was lower than non-low-income students (M = 7.25, SD = 2.08, *t*(127) = 2.57, *p* = 0.0113). In addition, the effect can be classified as medium-sized (*d* = 0.55), according to Cohen (1988). Taking into consideration the students who attended virtual lessons, again non-low-income students (M = 7.66, SD = 1.89) seem to outperform low-income students (M = 6.73, SD = 2.02, *t*(86) = 1.70, *p* = 0.0923). Due to the small number of low-income participants who participated in the virtual lessons, the difference is statistically significant if the confidence level is established at 90%. However, the effect (*d* = 0.48) should be considered as medium-sized too, in the same way as in the comparison with all students.

Secondly, we evaluated the existence of an income gap in the students’ performance when solving word problems using the intelligent tutoring system HINTS. To assess this eventual income gap, we used two measurements, namely, the number of problems solved during the teaching sequence and the ratio of problems solved correctly. Table 3 summarizes the average number of problems addressed in the virtual lessons and an average ratio score computed as the mean of the number of problems solved correctly, divided by the number of problems addressed by each student.

**Table 3:** Students’ performance when solving word problems in HINTS

Income	*n*	Problems addressed		Problem score ratio	
		M	SD	M	SD
Low	15	14.5	8.73	0.60	0.23
Non-low	74	17.1	8.81	0.65	0.24

An independent *t* test revealed that the average number of problems addressed by low-income students during the virtual teaching sequence was considered not to be significantly different from the average number of problems addressed by non-low-income students (*t*(87) = 1.04, *p* = 0.8502, *d* = 0.30). In the same vein, no statistically significant differences were found when comparing the score ratio of problems solved correctly between non-low-income and low-income participants (*t*(87) = 0.74, *p* = 0.4609, *d* = 0.21). In both cases, the effect sizes can be considered as small.

## Discussion and conclusions

In this paper we present a systematic review that we conducted of journals indexed in the Web of Science database with a focus on mathematics education. We may conclude from this study that research on mathematics in technology-based situations without teacher intervention is almost non-existent. Furthermore, the analysis of the distribution by educational stages of the reviewed articles revealed that a large part of the research concerning technology in mathematics is carried out with either pre-service or in-service teachers, and that studies on compulsory education are underrepresented. Likewise, it is also noteworthy that the qualitative research methodology is the one most widely adopted. Consequently, the results offer solid evidence that research in technology-based mathematics education has mainly opted for qualitative methods, targeted non-compulsory education and focused on proposals mediated by the teacher and developed synchronously. This could provide an explanation for the difficulty observed in different studies (Contini et al., 2021; Engelbrecht et al., 2020a, b) to address mathematics education under the singular circumstances posed by COVID-19.

Concerning the question of whether or not attendance in remote standalone instruction in COVID-19 times is conditioned by the income level of the students, the results obtained in the empirical example with HINTS are in line with other pieces of research (see, e.g., Andrew et al., 2020; Cullinane & Montacute, 2020), indicating that low-income students did not participate in online learning during the ERT situation as much as their non-low-income peers. Goldstein et al. (2020) pointed to two reasons to explain this phenomenon, namely, technology and adult supervision. Considering that all the students in our study were provided with the devices and internet connections necessary to be able to carry out the proposed activities, it seems plausible that parental supervision in this case would be the main cause of these attendance disparities. This concern was raised by Vekiri (2010), who suggested that supplying families with technological tools may not be sufficient to compensate for a lack of parental orientation. Bond and Bedenlier (2019) bore this in mind when they emphasized the importance of institutions conducting needs assessments (in addition to providing low-cost hardware and technology to students and families), in order to have a better understanding of the actual and potential barriers that students and families face, which we underline in this paper.

Regarding a possible income gap in mathematics academic performance during the COVID-19 lockdown, the fact that no differences were observed in the scores and SES levels of the participating students suggests that the use of HINTS would allow homogeneous support. These findings appear to be in conflict, to some extent, with those of Steenbergen-Hu and Cooper (2013), who previously pointed out a debate regarding how the use of technology would widen the achievement gap between different groups of students. In their meta-analysis, they found that the effectiveness of ITSs was lower for low-achieving students. On the contrary, Huang et al. (2016) concluded in a more recent study that ITSs can reduce the socioeconomic differences in mathematics performance. Despite the fact that the study was not conducted in the midst of a pandemic, it can be compared, in some measure, to the one presented here insofar as they share several characteristics, such as the participants' school stage, or the fact that they had access to the devices needed to work from home properly. In the case of our study, the students from disadvantaged backgrounds had lower grades in Mathematics before the school closure. However, during the online activities, students showed no statistically significant differences in mathematics performance, no matter their income level. The findings in the study by Engzell et al. (2021) during the school closures led to the conclusion that having access to technology is not enough to provide high-quality remote teaching, but our results suggest that things may be different in the case of ITSs.

This investigation has some limitations that allow us to make recommendations for further research. Regarding the systematic review, we focused our attention on journals with a specialization in mathematics education, but the review could be expanded to all journals, including those whose primary scope is technology. This would allow researchers to assess whether there is a gap between the research conducted in mathematics education and the technological opportunities available. With regard to the study carried out with HINTS as an example, for the measurements for poverty status, multi-national studies like PISA do not use administrative metrics such as free and reduced lunch-eligibility, since these metrics are generally not available and not comparable across countries (Avvisati, 2020). Although this issue would not arise in this study, the free and reduced lunch-eligibility measurement has been criticized for a number of reasons, including its sole reliance on income, the risk of misclassification, and the fact that it was not designed to be used as a measurement of low SES (Rutkowski et al., 2018). In addition, it would have been desirable to have a larger sample size and also a measurement of the students’ prior proficiency at solving word problems in a paper-and-pencil setting, before using HINTS. However, the lockdown situation offered limited possibilities and also made it impossible to gather such data.

Some advocate for one-to-one tutoring as a way to help struggling pupils to overcome COVID-19 learning losses (e.g., Kraft & Goldstein, 2020). Given the thesis that, in some cases, ITSs can be as useful as one-on-one human tuition (VanLehn, 2011), we also propose the use of ITSs with a fine granularity as a potential solution for mathematics education in general, and in ERT situations in particular, but more importantly, we consider it necessary to conduct more research in this area. Related to this, Jensen (2013) argued that providing students with positive, prompt, actionable, and task-specific feedback would help to narrow the gap in classroom engagement between low-income and non-low-income students over time. Hence, it is reasonable to expect that highly personalized feedback from an ITS would lead to similar outcomes, although this would need to be verified in longer-duration interventions. In any case, we would like to call attention to the fact that a third of students who were given the opportunity and technological means to engage in these online lessons with HINTS did not participate in even one lesson. This is consistent with estimates from other sources (e.g., Goldstein et al., 2020) and is something that any ITS, as well as any other platform, will find difficult to overcome, and which must be addressed as well by school leaders, policy makers and researchers. The evidence provided here can be taken into account not only when devising alternative or complementary methods to help students with arithmetical word problem solving, but also when looking for solutions when seeking high-quality mathematics education for all, even under the most adverse circumstances.

Research has always tried to respond to the needs of humanity and, in the case of education, the need for a standalone use of technology would not correspond to the *status quo* in the classrooms, where the teacher is a leading part of education. However, present and future technological advances require new ways of approaching mathematics education, and the recent pandemic events have done nothing but underscore this need. In this sense, it is surprising how little attention has been paid, in mathematics education, to the development of technological solutions based on artificial intelligence techniques. An intelligent system requires, among other aspects, the identification of the relevant actions of the students and their expression through specific knowledge of the didactics of mathematics. The scarce attention from the mathematics education community is surprising and could indicate the following: (a) basic research in mathematics education has not been transferred to real educational practices based on technology; or (b) research on technology applied to mathematics education has had a very limited vision, prioritizing a teacher-mediated use. These claims should not be understood as a complete amendment to the research carried out so far. However, this has resulted in a development and use of technology in the teaching of mathematics that has proven not to be sufficiently useful during the lockdown. Thus, before the pandemic, research focused on classroom settings and explored to what extent the use of digital technologies generates new ways of thinking about mathematics. As the emphasis has been placed on the possibility of using technology to do new mathematics, the possibility of using technology to do the same mathematics with a higher grade of students’ comfort and customization has been neglected. Perhaps the time has come to put aside the pessimistic view about the mathematics that students can learn when they tackle standalone tasks using technological tools.

## Data Availability

The data that support the findings of this study are available from the corresponding author upon reasonable request.
